# Subversion of Endoscopic Breast Reconstruction Surgery: Nipple-sparing Mastectomy and Immediate Reconstruction with a Latissimus Dorsi Flap/Latissimus Dorsi Flap and Implant Through a Single Axillary Incision

**DOI:** 10.1097/AS9.0000000000000082

**Published:** 2021-08-05

**Authors:** Faqing Liang, Nan Wen, Yanyan Xie, Yao Wang, Songbo Zhang, Qing Lv, Zhenggui Du

**Affiliations:** 1 From the Department of Breast Surgery, West China Hospital, Sichuan University, Chengdu, China.

## Abstract

Our team has created a novel endoscopic technique (ET) to solve the problems of the former minimally invasive surgery (Inconvenient operation for ET, high cost for robotic technique, long-operation time for both) for breast cancer patients requiring nipple-sparing mastectomy and immediate breast reconstruction (BR) with a latissimus dorsi flap/latissimus dorsi flap and implant. This easy-to-grasp method will benefit more patients.

## Abstract 2

Supplemental Digital Content is available in the text.

Unceasing efforts have been made in the development of endoscopic and robotic technology (ET and RT) to minimize the scar on the breast and back for patients undergoing BR with latissimus dorsi (LD) flap (LDF)/LDF and implant. However, surgeons were less enthusiastic about ET because of technical challenges.^1,2^ Although RT partly overcomes the disadvantages of the limitations of endoscopic instrumentation and difficulty in maintaining an optical window, it cannot be carried out as a routine procedure due to the high cost of the instruments.^3,4^ After 2 years of exploration, our team have created a novel ET to solve the problems mentioned above and reduce operation time, allowing surgeons to perform lymph node surgery, NSM, LD harvesting, and BR in the same operation just through a single axillary incision hidden in the armpit, which is first reported by whether by ET or RT.

## SURGICAL PROCEDURES

The patient was placed in a lateral decubitus position with the operative-sided arm available for axillary removal (see Video S1, http://links.lww.com/AOSO/A45). An axillary roll is used to prevent contralateral, brachial plexopathy. A 5–6 cm axillary incision was made. Axillary lymph node surgery was first performed under direct vision. Peng’s multifunctional operative dissector (PMOD) (Shuyou Surgical, Hangzhou, China) was used for the dissection of the retromammary space in a 3–5 cm area to obtain a working space. A 80-mm disposable wound protector (Surkon Medical, Wuxi, China) was placed through the incision and wrapped by the open end of one sterile surgical glove (7#) to seal the wound cavity. We inserted two or three trocars (5.5 mm*2 and 12.5 mm, Aesculap Inc, Center Valley, PA) through cut glove fingertips and fixed them with threads. CO_2_ insufflation was applied (12 mmHg) to maintain patency and sufficient optical cavity tension. Then, a coagulation hook (Aesculap Inc, Center Valley, PA) was used to dissect the entire retromammary space with the assistance of grasping forceps (Aesculap Inc, Center Valley, PA). In areas with abundant blood vessels, an ultrasonic scalpel (Ethicon Inc, Somerville, MA) was used. Then, in the subcutaneous plane, PMOD was used to release the upper outer quadrant of the flap to the retro-areolar tissue with open surgery. Moreover, a 0.5 cm skin incision (named “Huaxi hole 1” and allowed PMOD access) was created, located next to the areola in the upper-outer quadrant after reloading the protector and reinsufflation. Dissociation of the subcutaneous layer was continued using PMOD under endoscopy (KARL-STORZ Inc, El Segundo, CA) at the level of superficial fascia until mammary gland removal. For patients with thin subcutaneous fatty layer requiring BR with LDF and implant, the subpectoral space could be dissected before the retromammary space using coagulation hook, and the pectoralis major muscle was cut at the inframammary fold by ultrasonic scalpel. Subsequently, we unloaded the protector and switched to LD surgery. Similarly, the deep surface of the LD, 5–10 cm toward the spine and 18–20 cm toward the iliac bone, was dissected by PMOD under direct vision. Then, PMOD was inserted into a 0.5 cm skin incision (named “Huaxi hole 2”) and located 15 cm below the armpit, along the posterior axillary line, to complete the submuscular layer dissection. We used the same method to free the superficial surface of the LD. Then the LD was transposed to the front subcutaneous pocket, and two drains were inserted manually. The patient’s position was changed from lateral to supine for BR.

## OUTCOMES

We performed 8 NSM and immediate BR with LD/LD and implant (150–395 cc) by the novel ET without intraoperative conversion to open surgery for breast cancer patients, of which the mean BMI was 21.83 (range 19.90–23.44) kg/m^2^. The breast flap thickness was 0.5–1.2 cm. The operative time was 604 minutes for the first patient (with LD reconstruction) and decreased to 190 minutes for the most recent patient (with LD and implant reconstruction). Two patients had postoperative subcutaneous seroma in the back. Final pathology surgical margins of all cases were pathologically negative. No complications of bleeding, wound infection, flap necrosis, or axillary paraesthesia occurred. All patients were satisfied with the cosmetic effect. No local recurrence or distant metastasis was noted with a mean follow up of 7.75 (range 2–11) months.

## INNOVATION AND ADVANTAGES

1. The breast and LD were dissected from deep to superficial planes, opposite to the order in traditional surgery.^5^ Consequently, adequate air pressure propped up the superficial tissue like a tent, allowing adequate exposure of the operative field and facilitating excision and hemostasis without using various specific retractors.^6,7^
2. Using PMOD under endoscopy enables easier dissection of a dense tissue than coagulation hook (for ET) and monopolar cautery (for RT). Besides, PMOD inserted through “HUAXI hole 1” can move freely and easily reach the inner-lower, inner-upper, and outer-lower quadrants of the breast skin flap, which was difficult to achieve with endoscopic and robotic instruments. “HUAXI hole 2” overcomes difficulty of distant LD muscle resection near its paravertebral origin or iliac bone.
3. Compared with commercial gel-Port access, endoscopic instruments are more flexible with much cheaper and better-functioned self-made access, which partly overcoming the disadvantages of limited internal mobility and inadequate dissection angles, because trocars are not restricted by soft gloves and protector.
4. The whole procedure was completed within 190 minutes, which was much shorter than the reported RT completing such operation through a similar axillary incision and two notable small incisions (300 minutes).^8^ The RT was the only report of completing both NSM and LD harvest by minimally invasive therapy by far.
5. No obvious incision on or around the breast and back maximizes the esthetic outcomes and has any potential advantage in terms of an ischemic flap and wound infection.
6. The use of endoscope instead of a robot and self-made access rather than a gel-port make our surgical methods more economical and easier to popularize.

## CONCLUSION

The current satisfactory results demonstrate that our new technique is feasible, with a fine cosmetic effect. Further evaluation of the long-term results of this new technique will benefit more patients.

## Supplementary Material
